# 

**Published:** 1882-11

**Authors:** 


### CONTENTS.

***MISCELLANEOUS.*** PAGE

A "Regular's" Opinion of the Pseudo-Homœopath. Dr. Cathell, . . . 445

Pathological Materia Medica. Dr. Dunham, . . . . . . 446

The Presidential Address before the American Institute, 1882. Ad. Lippe, M. D., . . . . . . . . . . . . . . 447

Infinitesimals, etc. Dr. Fincke, . . . . . . . . . 453

Specifics. E. J. Lee, . . . . . . . . . . . . 456

The Silicea Headache. Dr. Dunham, . . . . . . . . 459

Lachesis and Lycopodium in Diphtheria. E. J. Lee, . . . . . 460

Fresh Air: A Necessity. Dr. Oswald, . . . . . . . . 461

Morphia; Hypodermically. J. Graball, . . . . . . . . 463

***CLINICAL BUREAU.***

Clinical Cases. E. P. Gregory, M. D., . . . . . . . . 464

Lycopodium Clinical Cases. C. W. Butler, M. D., . . . . . 465

Clinical Cases. E. W. Berridge, M. D., . . . . . . . 466

Supra-orbital Neuralgia. J. E. Linnell, M. D., . . . . . . 467

Clinical Cases. George G. Gale, C. M., M. D., . . . . . 468

A Clinical Case. J. W. Thompson, M. D., . . . . . . . 469

Lyc. in Bronchitis. Dr. F. Bruns, M. D., . . . . . . . 470

***BOOK NOTICES.***

Gatchell: "Doctor, What Shall I Eat?" Duncan Brothers, . . . 471
Hills: Observations on the Therapeutic Use of Alcohol. Reprint *Medical Times*, . . . . . . . . . . . . . 471
Morgan: Presidential Address. Reprint *Penna. State Society Trans.*, . 471
Faulkner: The Homœopathic Physician's Visiting List. Bœricke & Tafel, . . . . . . . . . . . . . . 472
Clapp: Catalogue and Price Current. Otis Clapp & Son, . . . . 472
NOTES AND NOTICES, . . . . . . . . . . . . 472

***APPENDIX.***

Cough Symptoms, . . . . . . . . . . . . 37–40
Hæmorrhoids. Dr. Guernsey, . . . . . . . . . 1–16

